# Epidemiological characteristics and public health responses against measles in the Ottoman empire and the early Turkish Republic

**DOI:** 10.1186/s12889-025-25833-z

**Published:** 2026-01-08

**Authors:** Ayşe Erkmen, N. Tüzün, O Erkmen

**Affiliations:** 1 Atatürk Principles and Revolution History Department, Gaziantep University, Gaziantep, 27310 Turkey; 2 Department of History, Faculty of Human Sciences, Ardahan University, Ardahan, 75000 Turkey; 3 Department of Nutrition and Dietetics, Faculty of Health Sciences, Istanbul Okan University, Istanbul, 34959 Turkey

**Keywords:** Measles, Measles morbillivirus, Ottoman empire, Public health, Archive document, Community impact

## Abstract

**Background:**

Measles is an infectious disease that affects mostly children. It is caused by the measles morbillivirus, which can be spread through inhalation and direct contact with the patient. There are insufficient literary documents on the prevalence of measles in the Ottoman Empire; its seasonal variation, the public health responses and epidemiological changes it caused.

**Methods:**

This study reviews archival documents from the Ottoman Empire and the Turkish Republic Archives, alongside relevant literature, to investigate measles cases, mortality patterns, seasonal distribution and public health interventions between the 18th century and 1938. The analysis focuses on quantifiable epidemiological data and recorded official measures such as quarantine, school closures, disinfection practices, and isolation protocols.

**Results:**

A total of 2716 people had measles in the Ottoman Empire, 462 (17.0%) of whom died. Among the patients, 116 (4.3%) were adults, and 2600 (95.7%) were children. Thirty-six (31.0%) of the adult patients died, and 396 (15.2%) of the child patients died. These figures represent only the documented portion that reached central administration through archival notification; the true burden in the Ottoman period was undoubtedly higher due to under-reporting and non-standardized disease registration, exacerbated by almost continuous warfare, territorial losses, population displacements, and administrative disruptions during the late nineteenth and early twentieth centuries, which further limited the completeness and continuity of public health reporting. In the early Turkish Republic (from 1920 to 1938), 67,427 measles patients were identified. Of these, 67,427 were children, and 4571 (6.8%) of the children died. The highest number of deaths from measles disease in Istanbul occurred in children aged 0–5 years (35.7%) in the Ottoman Empire.

**Conclusions:**

Measles was a persistent and high-mortality childhood disease in both periods studied with marked seasonal and regional variations. Archival records demonstrate that the Ottoman Empire and the early Turkish Republic implemented a range of organized public health responses to limit transmission, many of which are comparable to modern infectious disease control practices. This historical analysis may inform current epidemic preparedness by highlighting early institutional and community-level interventions.

## Background

Measles is an infectious disease caused by *Measles morbillivirus*, which can be spread through inhalation and direct contact with the patient. *M. morbillivirus* is a single-stranded, enveloped and segmentless RNA virus belonging to the *Measles* genus of the Paramyxoviridae family. Measles usually causes epidemics every 3–4 years, resulting in the development of non-immune cases [1, 2]. Measles was first described by Abu Bakr Al-Razi in 910 in his book “Al-Juderi wa’l-Hasbe”, stating that smallpox and measles were separate diseases and named as “hasaba”. This book was translated into more than a dozen languages [3]. This description by Al-Razi, one of the outstanding physicians of the Middle Ages, was a turning point in understanding measles and had a lasting impact on subsequent medicine. In 1757, it was reported to be caused by an infectious agent in patients’ blood [3]. In 1911, the measles agent was a virus [4], and the virus was first identified in 1954 [3]. During the Middle Ages, measles was an endemic disease in the Middle East, Africa, Asia and Europe [5]. In the 16th century, as global exploration increased, measles became more widespread, and in the 18th and 19th centuries, it became a deadly virus that could spread easily. Measles outbreaks devastated isolated communities such as the Faroe Islands in 1846, Hawaii in 1848, Fiji in 1875 and Rotuma in 1911 [6].

The measles vaccine was first produced and introduced in the USA in 1963 [3]. Although measles caused an estimated 2.6 million deaths worldwide each year before 1980, accelerated global vaccination programs reduced deaths; however, despite the vaccine, 145 thousand deaths from measles occurred worldwide in 2013. In 2017, 173 thousand measles cases were reported, rising to 229 thousand in 2018 [2]. In 2021, there were approximately 9 million cases and 128 thousand deaths from measles worldwide [7]. In Europe, 104,475 measles cases were reported from 2005 to 2008 [8],. In Turkey, 2904 people were infected with measles in 2019 [9]. Research on the seasonal prevalence of measles and the impact of seasons is insufficient [1]. The incidence of measles, which has been increasing in recent years, has shown that it may have invasive potential that can profoundly affect the global health burden and the lives of communities worldwide [1, 7]. For these reasons, outbreaks in different periods of history can be remembered, lessons can be taken, and more conscious behavior can be attempted against epidemics. Although we have the vaccine and health information to prevent measles, we are still far from eliminating the disease. Due to the rapid spread of measles disease and the fact that people in today’s world live and work more collectively than ever before, it may be necessary to remember the past epidemics and relevant precautions. There are insufficient literary documents on the prevalence of measles in the Ottoman Empire, its seasonal variation, and public health responses and epidemiological changes it caused. This study aims to examine the prevalence, demographic distribution, and seasonal variations of measles, and to review the public health measures implemented in the Ottoman Empire and the early Turkish Republic based on archival and literature sources. Precautions taken to control the disease have also been reviewed and evaluated.

## Method

The research was conducted between September 1, 2022, and May 30, 2024. A combination of digital archival searches was employed in the BOA (Ottoman Archives of the Presidency of the Republic of Turkey, Directorate of State Archives) and the BCA (Republic Archives of the Presidency of the Republic of Turkey, Directorate of State Archives). The BOA preserves correspondence and records (such as agreements, decrees, decisions, public health notifications, and epidemic reports) written in Ottoman Turkish using the Arabic alphabet from the early years of the Ottoman Empire until its dissolution. Comparable records from the Early Turkish Republic period (1920–1938) are preserved in the BCA.

**Fig. 1:**
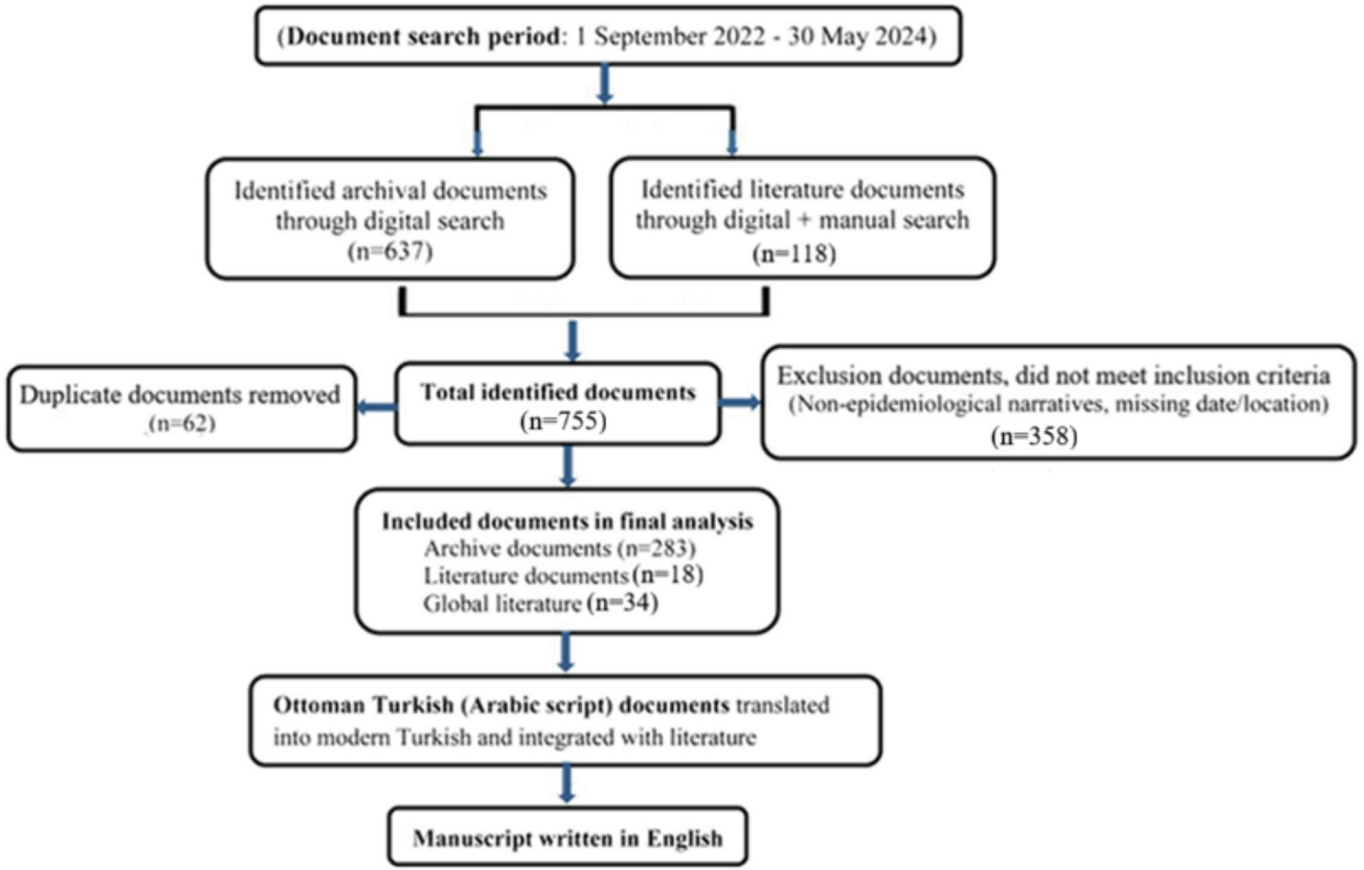
Flow diagram of document search, selection, and manuscript preparation process

Digital searches were carried out in the BOA, BCA, and newspaper digital catalogs via the official e-government portal (https://katalog.devletarsivleri.gov.tr/) using primary English (and Turkish) keywords: “measles” (“kızamık”), “hasaba,” “humma-i measles” (“kızamık humması”), “zymotic disease” (“bulaşıcı hastalık”), “morbilli,” “rubeola,” “red rash” (“kırmızı döküntü”), “febris rubeola” (“kızamık ateşi”), “rash fever” (“döküntülü ateş”), “measles outbreak” (“kızamık salgını”), and “childhood exanthema” (“çocukluk çağı döküntüsü”). Access to archival documents required official membership approval and a per-document fee for online viewing, by the regulations of the Presidency of the State Archives. The flow diagram of the document search, selection, and manuscript preparation process is presented in Fig. 1.

In addition to archival catalog searches, database searches (PubMed, Web of Science, Scopus, Google Scholar, etc.) were conducted using the same keywords. Manual literature searches were also performed, including the review of printed sources (books, journal archives, and yearbooks available in libraries) and reference list searches (identifying additional sources through the bibliographies of relevant articles, i.e., the “snowballing” method). This combined approach was necessary to retrieve materials not yet digitized or indexed under modern terminology but containing valuable epidemiological data.

Documents reporting measles alone or measles in conjunction with other diseases were identified and evaluated. Initially, 637 archival documents and 124 literature sources were identified. Duplicate records (*n* = 62) were removed. Subsequently, 364 documents not meeting the predefined inclusion criteria were eliminated. In the final stage, 283 archival documents and 46 literature sources were included in the analysis. Documents were classified into four groups (Fig. 1). BOA documents (*n* = 249) refer to primary sources from the Ottoman Empire period, obtained from archival records. BCA documents (*n* = 34) refer to primary sources from the Early Turkish Republic period, obtained from archival records. Literature documents (*n* = 18) represent secondary sources such as scholarly publications, reports, and historical studies concerning the Ottoman and the Early Republic periods. Global literature includes (*n* = 34) sources providing information on the course of measles, its epidemiology, vaccination policies, and related topics worldwide (e.g., WHO reports, international articles, and global statistics).

Archival documents related to measles, written in the Arabic alphabet in Ottoman Turkish, were transcribed and supplemented with references obtained from the literature review. Based on these sources, the present article on measles in the Ottoman Empire and the Early Turkish Republic (1920–1938) was prepared.

Inclusion criteria comprised documents that directly referenced measles, provided epidemiological data, described public health interventions, or contained mortality/morbidity records, and originated from the Ottoman Empire or the Early Turkish Republic.

Exclusion criteria comprised non-epidemiological narratives (e.g., purely literary or memoir-style accounts), documents lacking date or location information, and duplicate copies or reprints of documents already included in the analysis.

Study limitations should be acknowledged. First, archival research depends on the availability and preservation of documents; some records may have been lost, destroyed, or never recorded, leading to potential gaps in the data. Second, historical limitations in medical knowledge and diagnostic criteria may have led to alternative terminology or grouping of measles with other febrile exanthematous diseases. Although alternative historical terms were included in the search strategy, some cases may still have been missed or misclassified. Third, the scope of this study was limited to the BOA and BCA archives and literature documents; other relevant archives or unofficial records were beyond its scope. Finally, this is a descriptive historical epidemiological study. The findings provide insights into the epidemiology of measles and public health measures of the time, but do not establish causal relationships. Future research could expand the dataset by incorporating additional archival sources, triangulating data from international collections, or applying quantitative analyses where historical records permit.

## Results

**Fig. 2:**
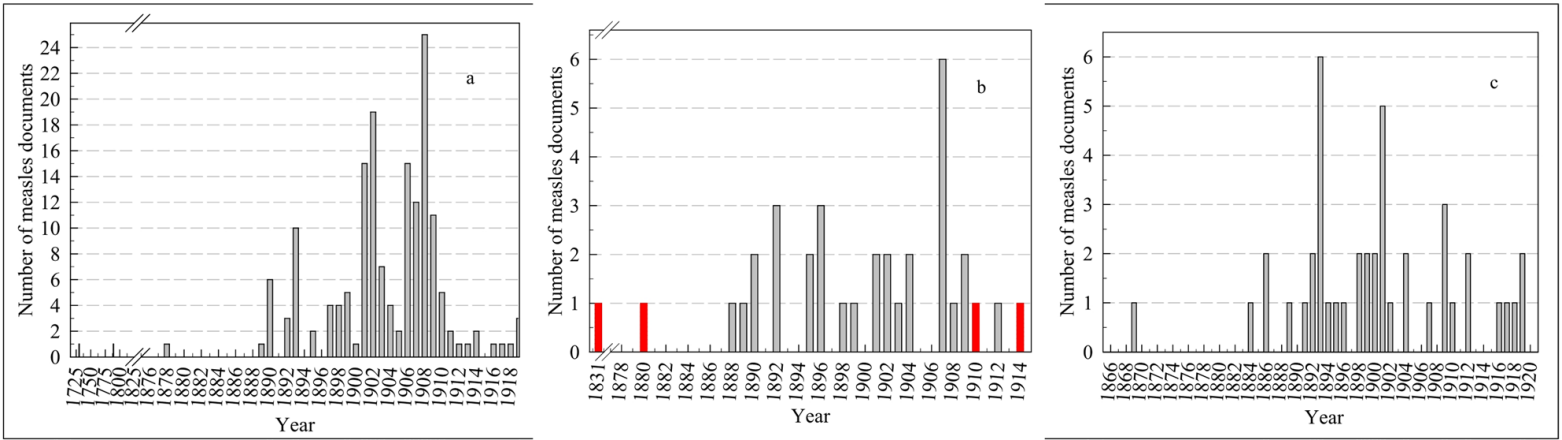
Number of measles-related documents in the Ottoman Empire from (**a**) Istanbul, (**b**) the Balkans and the Middle East (red), and (**c**) Anatolia. (Documents from Aleppo and Damascus in the Middle East, dated 1957, were not included in this figure) 999

**Fig. 3:**
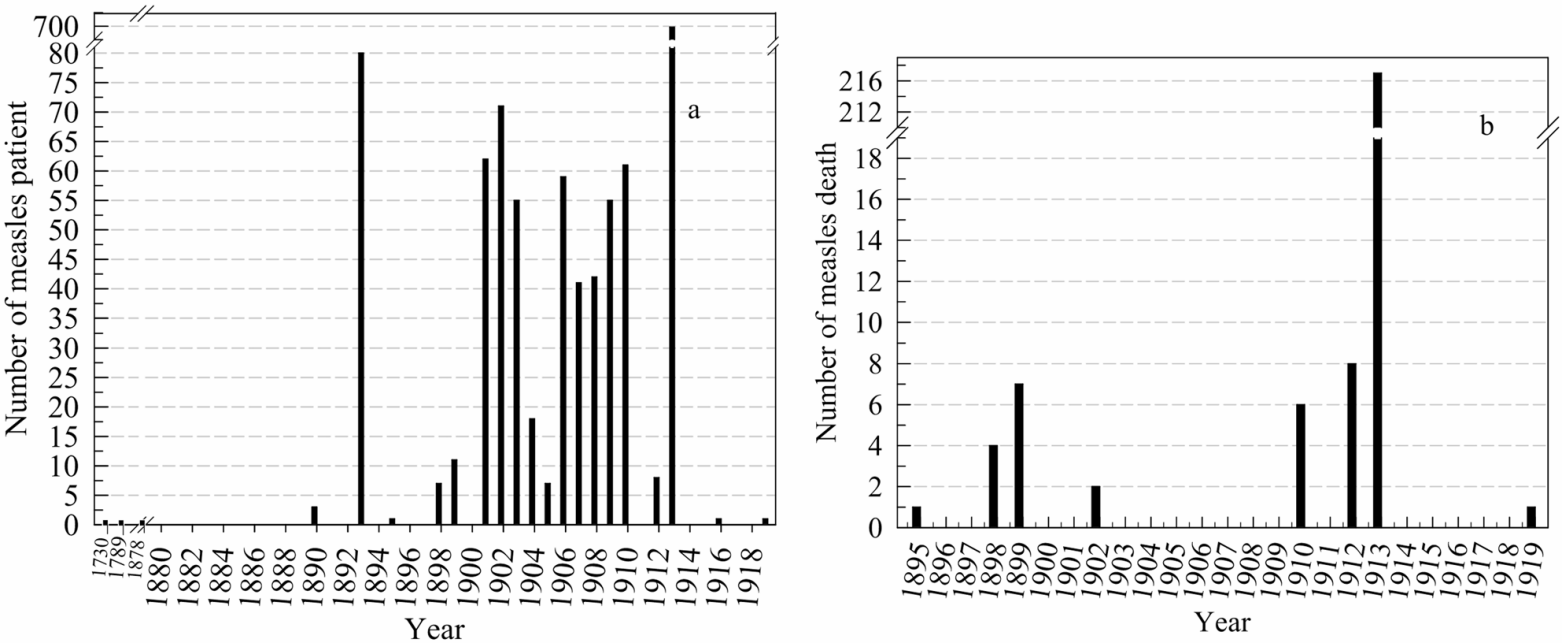
Measles disease (**a**) and death (**b**) in the Ottoman Empire depending on the year in Istanbul

**Fig. 4:**
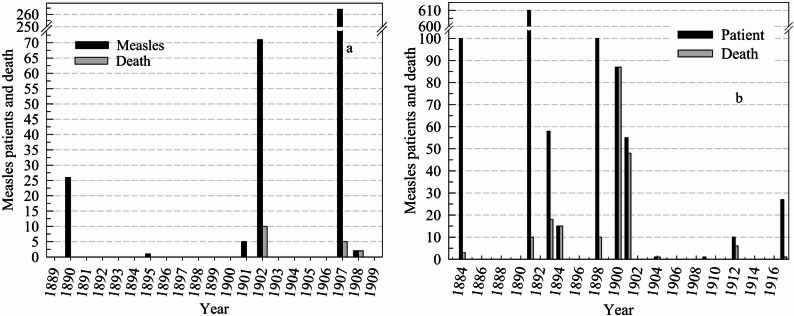
Measles disease and death in the Ottoman Empire depending on the year, in the Balkans (**a**) and Anatolia (**b**)

**Fig. 5:**
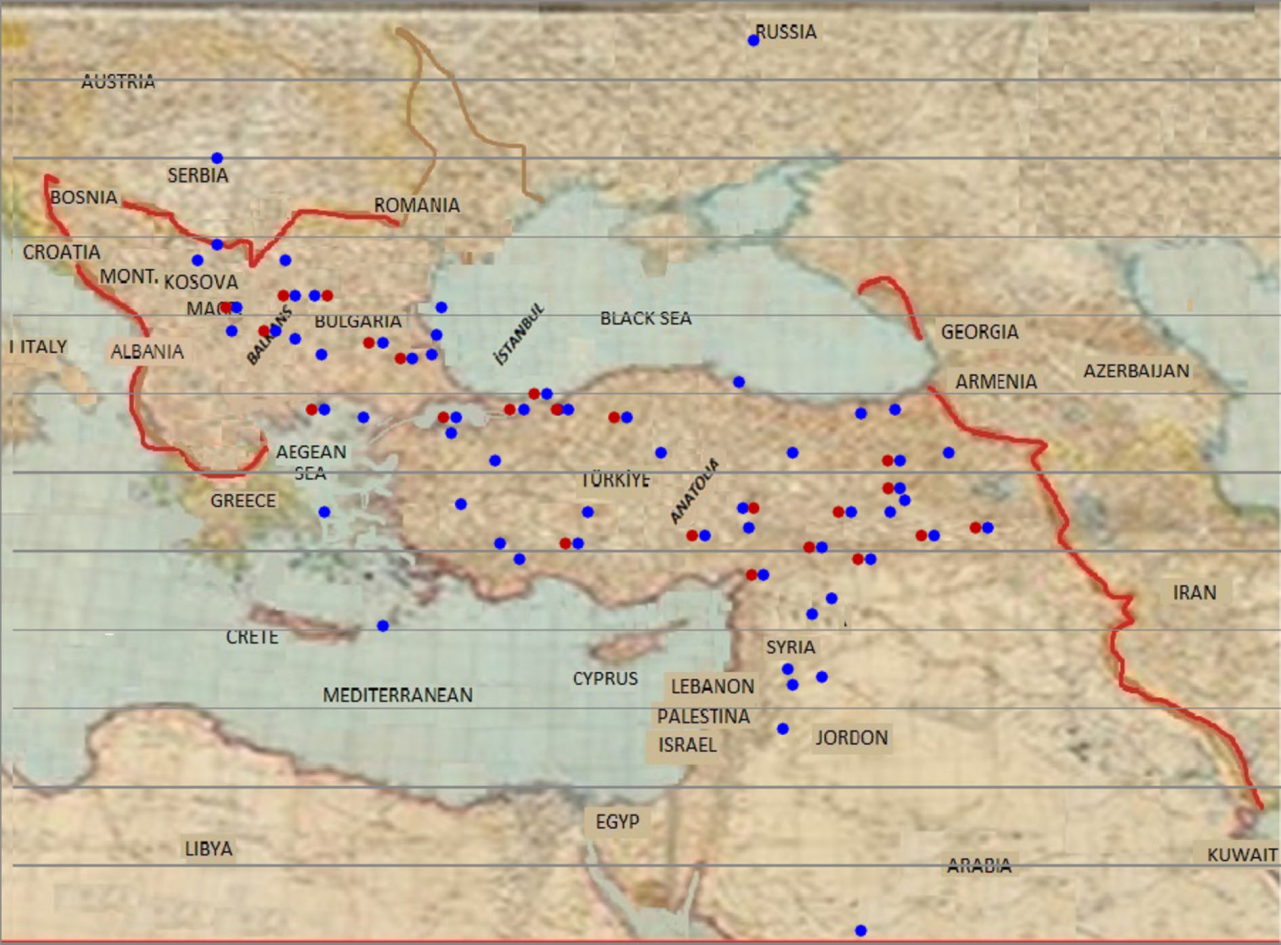
Map showing measles occurrence and deaths in the Ottoman Empire (except Russia and Serbia). Blue circles indicate locations where measles cases were reported; red circles indicate locations where measles-related deaths were recorded

This study investigated the status of the epidemic, public health responses and the precautions taken against measles disease in the archives of the Ottoman Empire and the early Turkish Republic. A total of 283 archival documents related to measles were identified. Of these, 240 pertain to the Ottoman Empire, 34 to the Early Turkish Republic, 6 to the Ottoman Middle East region, and 3 to states outside the Ottoman Empire (obtained from Ottoman Empire archives) (Fig. 2). Based on the surviving archival documents, a total of 2716 measles cases were identified for the years for which relevant records were available, and 462 (17.0%) of these cases resulted in death (Figs. 3 and 4) during the Ottoman period (1730–1919). Thirty-six documents from the early Turkish Republic were evaluated. Of these, 34 (94.4%) were archival documents, and two (5.6%) were literature documents. During the early Turkish Republic, 67,427 measles patients were identified, 4571 (6.8%) of whom died (Table 1). In addition, in two documents, only the number of deaths from measles in the Ottoman Empire was reported, indicating that 1939 people died of measles in Istanbul between 1899 and 1914 [9, 10]. The map in Fig. 5 illustrates the spatial distribution of measles and associated deaths across the Ottoman Empire.

**Table 1:** Number of measles patients and deaths among children1 in the Turkish Republic by year

Year	Patient	Death	Year	Patient	Death
1920	3	1	1930	2033	72
1921	142	18	1931	2863	146
1922	150	4	1932	4956	293
1923	242	10	1933	11,876	1000
1924	109	1	1934	5345	395
1925	2907	163	1935	4227	273
1926	3800	314	1936	6387	437
1927	2572	340	1937	5272	217
1928	3242	234	1938	8293	602
1929	3008	197	-	-	-
Total				67,427	4571

^1^Only one adult got measles in 1924 and 1925

The documents indicate that measles occurred in conjunction with other diseases, which are presented in Table 2. The number of documents reporting the number of measles patients in conjunction with other diseases is nine for Istanbul, one for Anatolia, and three for the Turkish Republic.

**Table 2:** Archival documents reporting the co-occurrence of multiple diseases^1^

Document	Diseases reported	Number of cases
BOA documents		
Two Istanbul documents	Measles, scarlet fever, diphtheria, chickenpox	5,8,1,1
Two Istanbul documents	Measles, diphtheria, scarlet fever	9,1,8
One Istanbul documents	Measles, scarlet fever	2,1; 5,1; 4,3; 2,1
One Istanbul document	Measles, mumps	8,1
One Istanbul document	Measles, chickenpox	5,1
One Istanbul document	Measles, smallpox	3,1
One Anatolia document	Measles, smallpox	7,6
BCA documents		-
One Turkish Republic	Measles, typhoid	3,2
Two Turkish Republic	Measles, whooping cough	1,2
One Turkish Republic	Measles, mumps, smallpox	1,2,2

^1^It was stated in 41 documents that different diseases occurred simultaneously with measles, without specifying the number of patients

## Discussion

In 1846, over 75% of the 7,782 inhabitants of the Faroe Islands reported having had measles disease [11]. In the USA, the mortality rate from measles was 1.8% between 1870 and 1879 [12], and 26 deaths per 1,000 measles cases were reported between 1912 and 1916 [11]. In Australia, the mortality rate from measles between 1878 and 1912 was reported as 6.4% [13]. In 1900, the number of deaths from measles and rubella in Prussia was 6,803 [14]. The number of measles patients and deaths recorded in the Ottoman Empire were 2716 and 462 respectively. These comparisons suggest that although measles was a global epidemic, its impact varied across regions depending on social and environmental conditions.

The relatively high mortality rates observed in the Ottoman Empire can be explained by political and economic instability, recurrent wars, and poor living conditions that created favorable environments for infectious diseases (BOA, DH.MKT./1280-92). Especially in Anatolia, poverty and the inadequacy of health services increased the vulnerability of the population. In the early Turkish Republic, the mortality rate (6.8%) was also higher than the world average. This situation can be attributed to the consequences of the First World War and the occupation of Anatolia and Istanbul between 1918 and 1922, which left the population in poverty and unhealthy conditions that persisted for years [15, 16].

Archival records also indicate measles cases among royal families outside the Ottoman Empire. For example, in 1887, the Emperor of Russia and his family were reported to have contracted measles, which delayed their stay in Copenhagen (BOA, HR.SYS./1342-58). In 1895, the eldest son of the King of Greece contracted measles but recovered without complications (BOA, Y.A.HUS./322 − 96). In 1898, King Aleksandra of Serbia was also reported to have measles (BOA, Y.PRK.ASK./17–31). These examples highlight that measles affected not only common populations but also royal families across Europe, underlining its widespread prevalence and social reach.

The comparative evidence from different regions emphasizes that mortality and morbidity from measles were shaped by a complex interplay of factors, including economic conditions, war-related disruptions, and the availability of health infrastructure. The findings from the Ottoman Empire and the early Turkish Republic therefore contribute to the broader understanding of measles epidemiology in pre-vaccination societies, providing insights into how political, social, and environmental determinants influenced epidemic outcomes.

### Earliest recorded measles cases

In the Ottoman Empire, the first recorded case of measles (“kızamık” in Turkish) appeared in archival documents in 1730 (BOA, HAT./1447-41). In this document, a woman from the Sultan’s family, presumably the daughter of Sultan Ahmet III in the Ottoman Palace, fell ill with measles and did not eat anything for four or five days. In 1789, the son of an Ottoman grand vizier was reported to have contracted measles (BOA, TS.MA.e./806 − 59). The first case of measles in the Middle East was recorded in Aleppo in 1757 [17]. The first measles detected in Anatolia during the Ottoman Empire was dated to 1869 [18]. The first document on measles in the Balkans, dated 1888 in Kochana, Macedonia, stated that children with measles were recovering (BOA, YB.021/83 − 55). The dates mentioned here represent the earliest cases we identified in surviving archival or published sources that clearly recorded measles in various regions of the Ottoman Empire; these dates do not imply that measles was absent in these regions prior to the years documented. The first report of measles in the early Turkish Republic, dated 1920, recorded three cases in a settlement, one of which resulted in death [15].

The first recorded death from measles in Anatolia during the Ottoman Empire occurred in 1884 in the Karamürsel district of Kocaeli (BOA, Y.PRK.ŞH./2–9), where a measles outbreak lasting for a week affected at least 100 children, resulting in three deaths, and prompted the implementation of cleaning, hygiene, and medical precautions. The first recorded death from measles in the Balkans occurred in Bulgaria in 1890 (BOA, Y.PRK.ASK./181 − 78), where 40 soldiers stationed in Bulgarian positions contracted the disease, 10 of whom died, leading to the implementation of hygienic and medical measures to prevent its spread. In the Ottoman Empire, the first recorded death from measles in Istanbul occurred in 1898 (BOA, BEO/1064–79759).

### Regional differences

According to archival records, measles was widespread in the Ottoman Empire between 1878 and 1919 in all regions except North Africa (Fig. 5). In 1910, it was reported that measles and smallpox were prevalent in the district of Khaybar (Medine), and that 250 doses of vaccine were needed (BOA, DH.MUİ./113 − 45). The number of measles patients (47.2%) and deaths (19.2%) was higher in Istanbul than elsewhere. The measles (39.2%) and mortality (18.7%) in Anatolia are much higher than those in the Balkans (13.6% and 4.6% respectively). In Anatolia, except for military service, there is little public circulation. Owing to poverty in Anatolia, especially in the early 20th century, people tried to survive in unhealthy conditions. These conditions create a favorable environment for the spread of infectious diseases [16]. In Anatolia, transportation and commercial exchange were minimal between different cities, and this circulation remained local in towns and villages. In this sense, compared to those in the Balkans, people in Anatolia were relatively isolated. When isolated communities encounter pathogens such as measles, people of all ages are affected, but children are particularly affected [12]. Owing to the effects of extreme isolation, the influenza pandemic killed many Pacific Island populations in the 1918–1920 [12]. At the beginning of the 20th century, the most important determinant of the rapid decline in measles mortality rates worldwide was globalization. Because of land and sea transportation network expansion, previously isolated populations were connected and integrated with the global community [2, 16]. In the mid-19th century, during military mobilizations in the USA, high mortality from measles was reported, especially among those recruited from rural areas [12]. The mortality rate from measles among US soldiers was found to be relatively low compared with that among Rotumans and South Africans. It was reported that this was because the society where the USA soldiers came from was not isolated compared with the Rotumans and South Africans [19]. In our study, recruitment from isolated communities was the reason for the high mortality from measles among soldiers. Research has shown that isolated life, health and environmental conditions, age and nutritional status, which are regional differences, play essential roles in measles disease and mortality.

### Seasonal differences

In the archives of the Ottoman Empire, the month and year are mentioned in only 95 archival documents about measles disease and those who died from this disease. In these documents, a total of 1785 measles patients were indicated, and 189 (10.6%) of them died. In the summer, 115 (6.4%) patients were sick with measles, and 8 (7.0%) of them died. In the fall, 121 (6.8%) patients were ill with measles, and 10 (8.3%) of them died. In the winter, 261 (14.6%) people were sick with measles, and 109 (41.8%) died. In the spring of 1288 (72.2%), people were seen with measles, and 62 (4.8%) of them died. The distribution of cases by month in the surviving documents suggests a possible seasonal pattern; however, these data represent opportunistic archival records rather than systematic surveillance across the entire Ottoman geography, and seasonal differences must therefore be interpreted with caution. Cold-related illnesses and the fact that the population lived in larger family groups (especially in Anatolia) likely increased the lethality of measles during the winter. In temperate climates, measles outbreaks usually occur in late winter and early spring [20]. While both hot and cold weather cause a decrease in the number of measles patients, relatively low humidity is a risk factor for measles deaths. There may be an increase in measles cases before and after cold weather conditions [21]. The study also revealed differences in the number of measles cases and deaths during the months of the seasons. In the Balkans and Istanbul, the highest number of measles disease cases occurred in April (258 and 150 patients, respectively), whereas in Anatolia, the highest number of measles disease cases occurred in March (645 patients). The highest number of measles cases occurred in March, the transition month from winter to spring. Deaths occurred in February (101 people), the last month of winter, and March (52 people), the beginning of spring. The study results showed that precautions to be taken against measles, such as vaccination, should be adjusted to account for seasonal variation, and the immunization of susceptible populations for measles elimination should be performed accordingly.

### Demographic structure

Among the 2716 measles cases in the Ottoman Empire, 116 (4.3%) were seen in adults, and 2600 (95.7%) in children. While 36 (31.0%) of the adult measles patients died, 426 (16.8%) of the child measles patients died. In Istanbul, 22 (1.7%) adults and 1260 (98.3%) children were infected with measles. Among these adult measles patients, 4 (18.2%) died, whereas 252 (20.0%) of the children’s measles patients died. In the Balkans, 72 (19.5%) adults and 298 (80.5%) children were infected with measles. In Anatolia, measles was reported in 22 (2.1%) adults and 1042 (97.9%) children; all adult patients died, whereas 184 (17.7%) of the children died. Since population denominators for each district were unavailable, these figures represent counts from surviving archival records and do not constitute population-based incidence rates.

The mortality rate from measles is relatively high in two regions (Istanbul and Anatolia) under investigation. In Istanbul and Anatolia, there were high rates of deaths from measles among children. In Istanbul, the high number of schools compared to those in the other two regions caused the measles disease to spread quickly. In Anatolia, there were problems due to the inadequacy of health conditions and health officials, especially in places such as the outskirts, neighborhoods, villages and towns. Studies have shown that diseases such as typhoid, scarlet, and measles became epidemic in Anatolia in 1869 due to the abundance of swamps, and in 1886 due to the lack of sewage facilities and contaminated drinking water [22]. For example, a document states that there was no doctor in Burdur Province; more than 600 children were sick with measles, and 10 of them died (BOA, DH.MKT/1823-8). Six people in Diyarbakır’s Eğil district were reported to be sick with measles, and 5 of them died (BOA, DH.MKT./126 − 37). Another study reported that 10% of measles patients in the Elbistan district of Kahramanmaraş died and that there was no doctor (BOA, DH.MKT/2088-12). The document in 1904 reported no doctors in Sivas and its districts; two doctors died due to the measles epidemic, and doctors were assigned from Erzincan [23].

In the Ottoman Empire’s archival documents and literature, only measles disease and deaths were reported according to school age in Istanbul. Among the 1260 measles children in Istanbul, 437 (34.7%) were aged 0–5 years, 626 (49.7%) were in primary school ages (6–10 years), 119 (9.7%) were in middle school ages (11–13 years), and 78 (6.4%) were high school ages (14–17 years). In Istanbul, 181 (41.4%) of children aged 0–5 years and 61 (33.7%) primary school children died. Children aged 0–5 years with weaker immunity were more susceptible to measles, and more deaths occurred. A high number of illnesses and deaths were also observed in primary school children, indicating that schools are effective in transmitting the disease agent. The low morbidity and absence of deaths in middle- and high-school-aged children may suggest that they are immune to the disease, probably because they have had measles at a young age. The results also indicated that social interactions that facilitate measles virus transmission occur when children come together in schools.

In only 13 archival documents in Istanbul, measles disease has been reported in two Armenian schools and 9 Greek schools belonging to non-Muslims of Ottoman nationalities, as well as in two French schools. An archival document of the Ottoman Empire specifies the nationality of measles patients in Anatolia (BOA, DH.TMIK.M./81 − 16). In this document, it is reported that 87 people of Ottoman Armenian nationality died of measles in the township of Dersim, without indicating the total number of cases; in 1900, 68 of the deceased were children. In Istanbul, only four archival documents in French and Greek schools and 5 Armenian schools stated that 114 children had measles (BOA, BEO./205–15336; BOA, MF.MKT/545-9; BOA, MF.MKT./651 − 35; BOA, MF.MKT./639 − 29). In 1893, in the villages of Milad and Bolazar in the Karasu subdistrict of Sakarya, 11 out of 30 measles patients died (BOA, DH.MKT./8–88). In 1901, in Evlek Village, Bitlis Province, 20 of 22 individuals infected with measles died (BOA, DH.MKT./2458 − 126). In 1890, in the town of Harmanlı (Bulgaria) the air temperature reached 35–36 °C, and a high number of deaths from scarlet fever and measles was reported (BOA, DH.MKT./178 − 49).

In the Ottoman Empire, measles was observed not only among the population but also among soldiers. It was reported that 40 of the Ottoman soldiers in Bulgaria had measles disease, 13 (32.5%) of whom died (BOA, Y.PRK.ASK./181 − 78); 31 soldiers had measles disease at the Bulgarian border (BOA, Y.PRK.ASK./181 − 85); and four soldiers died of measles in Istanbul and Elazığ (BOA, DH.MKT./2487-1; [24]). Similarly, During the American Civil War (1861–1865), approximately 67,000 soldiers contracted measles, and more than 4,000 of them died [25]. In the USA, measles outbreaks occurred among soldiers from 1917 to 1918; approximately 2,000 soldiers died, and most of the measles-related deaths among soldiers were caused by secondary bacterial diseases [19]. Recent historical scholarship has also shown how vaccination policies and public attitudes have shaped measles epidemiology in different national contexts [26, 27].

In the Ottoman Empire, the number of villages, towns, and districts in which measles was reported in archival documents outside the provinces was 54, and the number of settlements that can generally be considered outskirts in cities was approximately 27. In these archival documents, a total of 1602 measles patients were identified, 163 (10.2%) of them died. The number of measles patients in the provinces’ centers and economically better-off settlements was 284 (15.1%), of which 1.4% died. In underdeveloped and economically weak settlements, measles disease and mortality rates were high. In the Ottoman Empire, residents of towns and villages had inadequate access to healthcare. In the early Turkish Republic, settlements did not record measles disease in archival and literature documents.

In the Ottoman Empire, the sex of measles patients and deaths were not recorded outside Istanbul. However, in Istanbul, only 831 (66.0%) sex of measles patients were indicated. Four hundred twenty-three (50.9%) of them were female, and 408 (49.1%) of them were male. Among these patients, 27.4% and 24.8% died, respectively. During the early Turkish Republic period, very few documents recorded the sex of measles patients (162 measles, 0.2%). Only 135 (83.3%) were reported as girls, and 27 (16.7%) were reported as boys. When the Ottoman subjects were analyzed according to their beliefs, the measles data were as follows: Among the Muslim measles patients, 37 boys and 36 girls aged 0–5 years and one woman aged 21 years were identified. Among non-Muslim people, four boys and nine girls aged 0–5 years from the Greeks, one boy and one girl aged 6–20 years; two girls aged 0–5 years from the Armenians and one boy aged 6–20 years; and five boys and four girls aged 0–5 years, and three girls aged 6–20 years from the Jews were reported to have died. Gender is essential concerning measles death, and mortality rates may be higher in girls than in boys [25]. Our research also revealed that in 1914, girls had a higher rate of measles and died than boys did.

### Community impacts and public health measures

The archival evidence presented in this section includes both measles-specific responses and broader epidemic-control measures applied to multiple infectious diseases. In this revised version, the measles-specific interventions are noted explicitly, while general epidemic measures are indicated as such, so that the two categories are clearly distinguished.

Health services in the Ottoman Empire were generally fee-based. However, during epidemics, free treatment was occasionally provided for poor citizens. For example, in 1893, measles among children was reported in Urfa Province; municipal health officials implemented cleaning and disinfection precautions in schools and provided free treatment to poor families (BOA, DH.MKT/2055 − 114). Similarly, in 1912, records from Resne Township (Macedonia) noted that poor people with measles and smallpox should receive free treatment (BOA, DH.İD./136–19912). These examples illustrate how epidemics not only strained health systems but also prompted inclusive public health practices.

Measles epidemics also had significant economic and political consequences. Outbreaks were widespread in many regions of the Ottoman Empire (Fig. 5). Wars between 1683 and 1918, internal rebellions, poverty, and mass migrations accelerated the spread of measles and increased mortality (BOA, Y.PRK.ASK./181 − 78; BOA, DH.MUI./26–27; BOA, DH.MUI./70 − 6; BOA, Y.PRK.ŞH./2–9). Archival sources record that villages and neighborhoods were sometimes abandoned due to epidemics, while migrating populations carried the disease to new settlements, which in turn reduced tax revenues (BOA, A.DVN.MHM.d./79–768; BOA, MAD.d./14739-1065-11; BOA, İEDH./3–262). To prevent depopulation and economic disruption, the Ottoman administration occasionally granted tax exemptions or reductions in epidemic-affected regions (BOA, TSMA.d./1306-63; BOA, IE.SH./2–125). Epidemics, therefore, not only caused health crises but also weakened the fiscal base of the state, creating long-term economic and social difficulties [28].

Mass migrations also played a critical role in disease dynamics. Following the wars with Russia, between 5 and 7 million people migrated from Crimea and the Caucasus to Anatolia [29]. In the 19th century, migrations from the Caucasus deeply affected Ottoman demography. At the same time, 75.4% (1,757,101 people) of the Muslim Turkish population from the Balkans migrated to Istanbul and Anatolia between the late 19th century and 1918 [30, 31]. These migrations, combined with harsh economic conditions, poor hygiene, and malnutrition, created favorable environments for epidemics and increased measles mortality in Anatolia [16, 32].

The public health response was relatively organized in Ottoman Empire and Turkish Republic. Authorities implemented measures such as school closures, household isolation, disinfection of goods and buildings, and temporary suspension of community activities. Archival sources record closures of schools in Istanbul including Kadıköy Hamidiye and Erenköy Boys’ Secondary Schools, Esmahan Kaya Sultan and Kadırga Primary Schools (BOA, MF.MKT./1041-14; BOA, MF.MKT./553 − 12; BOA, MF.MKT./553 − 18). Schools were sometimes closed indefinitely, as in Yakub Ağa School (BOA, MF.MKT./572 − 58), while in other cases, reopening was permitted only after the epidemic subsided (BOA, MF.MKT./155 − 125). Cleaning and disinfection were conducted under municipal supervision, such as at the Greek Primary School in Kumkapı (BOA, MF.MKT./545-9).

Other preventive practices included isolation of infected individuals in homes and schools (BOA, MF.İBT./201 − 16; BOA, MF.İBT./204 − 113; BOA, MF.İBT./268 − 60), substitution of teachers when a teacher or their child became ill (BOA, MF.İBT./190 − 33; BOA, MF.İBT./204 − 113), and disinfection of households, schools, workplaces, cars, boats, letters, and packages by “*tebhirhane*” facilities using “*pulverizer*” machines [33]. Quarantine measures were also applied, such as the medical inspection of passengers arriving at Ottoman ports (1913) and trains from Austria-Hungary (1914) [34].

Despite these measures, public acceptance was inconsistent. Some groups resisted isolation and quarantine, viewing them as religiously inappropriate. Records describe uprisings, such as the 1840 Amasya incident, where quarantine officials were attacked and killed, and the 1845 revolt by Hejaz pilgrims in Adana who looted quarantine buildings [35]. In 1848, resistance was also reported in Gaziantep against the quarantine applied to travelers from Aleppo [35]. To counter opposition, the Ottoman government frequently sought religious legitimization; fatwas were issued to confirm that health measures were compatible with Sharia law [35].

### Public health and precautions taken

The Ottoman Empire took precautions, such as school vacations, to prevent the spread of measles when even one person in a school was infected. In the school where the disease was detected, the children were immediately sent home, and the necessary cleaning and hygiene precautions were requested to be taken in homes for sick children. Isolation was practiced at home with measles, and it was stated that the patient should not have been in contact with other family members and that the family should not have been in contact with others. In crowded places (such as schools, barracks, and hospitals), precautions were taken against diseases due to disease because of concerns that measles would become an epidemic. In Istanbul, Esmahan Kaya Sultan Primary School (BOA, MF.MKT./553 − 12) and Kadırga Primary School (BOA, MF.MKT./553 − 18) were closed for 12 days due to measles. From time to time, there were also cases where the opening date of schools was not specified. Yakub Ağa School in Istanbul was closed because of the measles outbreak (BOA, MF.MKT./572 − 58). In some neighborhoods of Eyüp in Istanbul, schools were suspended due to measles and scarlet fever in children, and it was stated that schools could be opened after the disease disappeared (BOA. MF.MKT./155 − 125). Sometimes, cleaning, disinfection, or renovation is carried out during epidemics. Owing to the increase in epidemic diseases, the Greek Primary School in Kumkapı (Istanbul) was suspended for cleaning under the supervision of municipal authorities (BOA, MF.MKT./545-9).

Health services, which were paid for in the Ottoman Empire, started to be provided free of charge in the Republic of Turkey. Precautions similar to those taken by the Ottoman Empire were also taken during the Republic of Turkey. During the Republican period, school vacations were used as a preventive precaution. Esma Sultan Girls’ and Kapudan Hasan Paşa Primary Schools in Istanbul had been suspended for 10 and 15 days, respectively, due to measles (BCA, 180-9-0-0/12–70-11; BCA, 180-9-0-0-0/12–70-14). In the Ottoman Empire, precautions were taken to ensure that the items collected and distributed for charity did not transmit and spread diseases. Decisions were made to ensure that items (such as used clothing and quilts) collected to aid needy people could not transmit measles when not cleaned and disinfected (BOA, DH.MKT./1280-92).

Health officials in the Ottoman Empire practiced isolation to prevent the spread of measles. Three primary school students in Istanbul had measles, and the disease was transmitted to another person; therefore, measles was contagious, and necessary isolation precautions were taken in schools and their homes (BOA, MF.İBT./201 − 16). When a teacher or a teacher’s child contracted measles, the teacher was given leave for the illness, and a substitute teacher was assigned (BAO, MF.İBT./190 − 33; BOA, MF.İBT./204 − 113). Authorities sent students who showed sneezing and watery eyes home, and cleaning and disinfection procedures were carried out in places (BOA, MF.İBT./268 − 60). Necessary isolation precautions were taken in places where the disease was observed and schools closed (BOA, MF.İBT./204 − 108; BOA, MF.İBT./207 − 96; BOA, MF.İBT./24–97).

Candy sugar (akide şekeri)^1^, which is thought to be beneficial for measles, is fed to measles patients. It was stated that measles disease was widespread in children in Niğde Township, that it was impossible to treat it and that it was essential to provide patients with sugar, but no sugar was available (BOA, DH.İ.UM.EK./105 − 100). It was requested that there was a great need for sugar used in treating measles in hospitals and that sugar be provided as soon as possible (BOA, DH.İ.UM.EK./105 − 100).

Public screenings concerning measles were conducted before epidemics to prevent the occurrence of the disease. In 1886, doctors and pharmacists were assigned to conduct screenings for measles in Erzincan Township and its villages, and state officials and residents were asked to assist health personnel during screening (BOA, DH.MKT./1404-76). In the case of insufficient medical personnel, people from health-related professions (such as pharmacists) were also assigned to fight against diseases. A pharmacist assigned as a health officer was awarded a medal (Order of Osmania) for his success in treating children suffering from measles in Istanbul (BOA, DH.MKT./339 − 11). When measles cases were observed in orphanages in Istanbul, activities in overcrowded orphanages were requested (BCA, 272-0-0–11/15–61-1).

The Ottoman Empire imposed quarantine to prevent the spread of epidemics when necessary. The quarantine precautions imposed in Istanbul, Bulgaria and against the ports on the European coast of the Aegean Sea were lifted on November 18, 1913. On November 19, 1913, it was announced that all arrivals to the ports would be subject to medical examination [34]. On October 15, 1914, it was announced that passenger trains arriving from Austria-Hungary would be subjected to medical examinations at the border.

In the Ottoman Empire, physicians wrote articles in magazines and newspapers to raise public awareness about measles disease, explain the precautions they had taken, and explain treatment practices. For example, in *The Wealth of Science and Technology Journal* (*Servet-i Fünun Dergisi*) in 1892, Physician Ömer [36] described the course of measles disease and the symptoms to be seen in the patient and explained the treatment of measles and the precautions for the disease to be taken. In his article, Ömer, after describing the necessity of ventilation and cleaning the room where the measles patient was located, stated that feeding the patient blackcurrant and cherry sherbet would be appropriate. Again, in this article, “*to speed up the process of the patient’s shedding of measles and to reduce coughing*,* a little brandy or rum was added to the juice made from fruits boiled in water*,* and the patient was asked to apply it to the patient’s back morning and evening for two days.”* It was stated that the patient should be fed a thick mixture of Ipeka prepared from the golden root (Ipeka, *Rhodiola rosea*) with a coffee spoon every half hour. If the disease is not relieved, a physician should be consulted. In an article by physician Hilmi [37] in the *Physician Journal*: *“The measles patient should be bathed in 20*,* 22 or 24 °C water. The patient’s nose*,* throat*,* and eyes should be washed with boric acid solution* (2–3% w/v), *and two teaspoons of castor oil* (bean, *Ricinus communis*) *should be given to the patient. If the patient breathes with wheezing and noise*,* they should be made to vomit with “vomiting syrup”.*^2^*One coffee spoon of syrup should be given every 10 minutes until the patient vomits. After vomiting*,* if the patient wishes*,* only milk and tea should be given to the patient”.* An article written in the *Magazine Idea (Mefküre)* [38] stated that *“Those who do not catch measles when they are young will catch it when they grow up”.* The article continues: *“Measles is a disease that is transmitted from one person to another. The patient’s food should consist of watery dishes. Milk*,* yogurt and meal soup should be given. They should drink water*,* lemonade and “red sherbet”* (mixture of cranberries, blackberries and cherries juices). *After the patient gets better*,* they should be given a bath*,* and the room where the patient stays should be ventilated*,* cleaned*,* and disinfected for 1–2 weeks*,” and he was advised to do so.

A regulation (“nizamname”) was promulgated in 1913 to specify the precautions to be taken in schools. In the event of a measles outbreak in schools, depending on the form and severity of the epidemic, medical inspectors, in consultation with the chief inspector, were given the duty to suspend schools and to ensure that the children did not attend school until they fully recovered. Students suffering from notifiable diseases (such as measles, whooping cough, chicken pox, baldness, scabies and tuberculosis) were prohibited from attending school. The rules for schoolchildren also apply to teachers and other employees [39].

In the Republic of Turkey, the Ministry of Health and the General Directorate of Medicine and Pharmacy were established in 1920. An institutionalized fight against diseases was ensured. Health services, which were paid for in the Ottoman Empire, started to be provided free of charge in the Republic of Turkey.

### Public response to the precautions taken

The administrators of the Ottoman Empire adopted a rational and scientific approach to combat epidemics. As a first precaution, quarantine and isolation precautions were taken. On the other hand, the public took a fatalistic approach to epidemics, reacted against quarantine and isolation to the point of rebellion and caused setbacks in taking precautions. The Ottoman Empire obtained fat from religious officials to prevent the public’s reaction and announced that the precautions were appropriate [33]. In 1893, when measles was seen in children at a French Primary School (Foreign Country School) in Istanbul, the school was asked to be closed until the disease was eliminated. However, the French school authorities defied this order and did not want to close the school, so it was reported that an investigation was opened against the school administrators and was closed (BOA, BEO./212–15851). In 1898, measles was reported in the French Girls’ Primary School (Foreign Country School) in Kadıköy (Istanbul), and the school administration prevented health officials from going to the school to examine the students. As a result of the negotiations between the Ministry of Foreign Affairs and the French Consulate, a representative from the consulate would come to the school, and the students would be examined in the presence of the representative (BOA, MF.MKT./387-9). It was also stated that an investigation was opened against municipal health officials in Istanbul who did not report croup, scarlet fever, measles, smallpox and typhoid fever to the Ministry of Health. An investigation was carried out against them, and fines were imposed due to the families’ opposition to the treatment and isolation practices that were to be carried out by the health officials in the houses (BOA, BEO./1064–79759).

During epidemics, clergypersons objected to quarantine practices by stating that quarantine was not religiously appropriate. This opposition sometimes turned into a popular uprising. In 1840, after the noon prayer at the Bayezit mosque in Amasya, the mosque’s teachers did not allow the mosque to leave the mosque and make speeches against the quarantine practice. They said that quarantine had no place in Sharia and that the physicians who practiced it should be fired immediately. With this provocation, people attacked the quarantine building, broke the door, and entered and killed the quarantine physician [35]. Owing to the epidemic in Hejaz, it was decided to impose quarantine on pilgrims from Hejaz in 1845 outside Adana as a precaution, and quarantine tents were set up for pilgrims. However, the pilgrims revolted against the quarantine practice and prevented it by looting the quarantine building [35]. In 1848, due to epidemic diseases in Aleppo Province, quarantine was imposed on travelers from Aleppo to Gaziantep. However, those quarantined broke down doors, windows, and quarantine flags of the quarantine building and tried to kill the quarantine director [35]. In the Ottoman Empire, the death of people working in agriculture, animal husbandry, trade, labor and other institutions due to diseases such as measles was a loss for the state in terms of production and services, and many expenses were incurred in the fight against the disease. In addition to causing a decline in the economy, this situation has caused deterioration in public health, deaths and unrest in society [35]. People rebelled against the physicians who examined those who died from epidemics and considered the physicians’ examination as a hand reaching for their honor. They also saw the precautions taken to prevent outbreaks as going against God’s will [35].

### Arrangement of education buildings

During epidemics, inspections were carried out mainly in crowded and communal areas. In case of need, public buildings were transformed into places that could provide health services. During the Balkan Wars (1912–1913), some schools were converted into hospitals due to epidemics (BOA, DH.SYS./112 − 20). In 1894, health inspectors increased their inspections in public places (such as schools, student dormitories, inns, guesthouses, mosques, dervish lodges, churches and workshops) to ensure the improvement of unsanitary conditions (BOA, Y.A.RES./72 − 1). The health standards in student dormitories in Istanbul, including the dormitories of the Military High School, were inadequate, and inspections were requested (BOA, İ.MF./2–19; BOA, İ.HUS./16–153). In the 1890 s, municipal doctors in Istanbul were instructed to visit dormitories for at least two hours a day to examine their children, pay attention to the school’s supervision, and report any situation they encountered during their duties to the center [40]. In 1894, the Ottoman Empire established a “Sanitary Commission” in Istanbul under the chairmanship of Miralay Bonkowski and charged it with the effective cleaning and disinfection of Istanbul. Bonkowski prepared reports on improving the unsanitary conditions in mosques, churches, synagogues, schools, prisons, hospitals and some houses (BOA, İ.HUS./1–12). In line with the commission’s reports, cemeteries were established outside the settlements for those who died from epidemics (BOA, İ.HUS./1–12).

Moreover, the relatively low number of measles cases detected for the Ottoman period reflects notification limitations rather than a low historical incidence. Disease reporting was not centrally standardized, population-based health registration did not exist as in the Early Republic, and most of the population considered illness as a divine fate rather than a reportable public event. Local authorities often did not transmit morbidity information to the capital and communication networks were fragmented and slow. Bilsel [41] also noted that the “true figures are at least twice, and with unreported and untreated cases probably three times higher than those recorded”. Thus, the 2716 cases reflect only the reported archival counts; the true disease burden must have been substantially higher. In the Early Turkish Republic, protective health services were institutionalized as a state policy, and new facilities for combating infectious diseases (such as malaria, trachoma, typhus, typhoid and tuberculosis) were established, including the Refik Saydam Hygiene Institute in Ankara which produced vaccines and sera [42]. Therefore, notification in 1923–1945 was substantially more standardized and likely more complete than in the Ottoman period. Furthermore, demographic comparisons across regions should be interpreted cautiously, as the surviving counts could not be adjusted for the population at risk in each location. Additionally, seasonal differences should be interpreted with caution, as the available archival cases do not constitute continuous time series and do not control for climatic variation across regions.

Historical medical scholarship indicates that pneumonia was the most frequent severe complication of measles and a major driver of measles mortality in the nineteenth and early twentieth centuries [43, 44]. In the Ottoman archival material examined here, the distinction between primary measles and fatal secondary complications such as pneumonia is not always explicit, reflecting the limited diagnostic capacity of the period. Consequently, some deaths recorded simply as “measles” likely include deaths due to measles-associated pneumonia or other complications. This diagnostic ambiguity is a recognized limitation of historical mortality statistics and further complicates the interpretation of cause-specific death counts.

In addition, the fragmentary nature of surviving archival sources necessarily creates uncertainty. The number of cases documented here reflects only those captured in preserved reports, and the magnitude of this undercount likely varied over time and space. In addition, retrospective diagnosis poses challenges, because the clinical definitions and disease categories used in the eighteenth, nineteenth and early twentieth centuries evolved continuously; a “measles” designation in a historical record may at times reflect a different febrile exanthematous disease. These issues highlight that such research requires an interdisciplinary approach, combining historical expertise with epidemiological methods. Our aim in this study was to demonstrate how such collaboration can enrich disease history, while also clarifying the interpretive limits of the surviving data.

## Conclusions

In the Ottoman Empire and the early Turkish Republic, measles was a frequent and high-mortality childhood disease, particularly among children aged 0–10 years. Archival records document significant regional and seasonal variations in incidence and mortality, with peaks in spring and highest mortality in winter. The majority of cases occurred in economically disadvantaged and rural areas, where inadequate health infrastructure, malnutrition, and poor hygiene conditions contributed to increased lethality. The subgroup analysis of available archival data indicates that certain professional groups, such as soldiers and students, as well as specific ethnic and religious communities, were disproportionately affected during some outbreaks. These findings highlight the heterogeneous impact of measles within the population and suggest that social and structural determinants of health played an important role in disease outcomes. Public health responses recorded in the archival sources included school closures, isolation of patients, disinfection of buildings and goods, and, in some cases, provision of free treatment to the poor. These measures, although constrained by the limited medical knowledge and resources of the time, demonstrate a structured and organized institutional response to epidemic control. Community resistance to some measures, such as quarantine, also illustrates the social challenges faced in implementing public health interventions. This historical analysis underlines that, despite the absence of vaccination, coordinated public health responses were implemented in both the Ottoman Empire and the early Turkish Republic. Lessons from these past experiences may inform modern epidemic preparedness, especially in resource-limited settings, by emphasizing the importance of early detection, targeted intervention in high-risk groups, and adaptation of measures to local socio-cultural contexts.

## Data Availability

No datasets were generated or analyzed during the current study.

## Footnotes

1 In Turkish “akide şekeri”, that is unique to Turkish cuisine. It is prepared as followings: The dough is obtained by boiling sugar, starch and water and then adding different sweeteners, colorants and flavorings. It is shaped using special molds, cooled and cut into small sizes.

2 Preparation of vomiting syrup: 0.5 g *Cephaelis ipecacuanhae* powder + 50 Ipecac syrup. Preperation of ipecac syrup: 10 g *C. ipecacuanhae* powder **+** 90 g Simple syrup 90 g. Preperation of simple syrup: 64 g Saccharoze + 36 g Water.
